# Association between air pollution in Lima and the high incidence of COVID-19: findings from a post hoc analysis

**DOI:** 10.1186/s12889-021-11232-7

**Published:** 2021-06-16

**Authors:** Bertha V. Vasquez-Apestegui, Enrique Parras-Garrido, Vilma Tapia, Valeria M. Paz-Aparicio, Jhojan P. Rojas, Odón R. Sanchez-Ccoyllo, Gustavo F. Gonzales

**Affiliations:** 1 High-Altitude Research Institute; Laboratories of Investigation and Development (LID), Department of Biological and Physiological Sciences, Faculty of Sciences and Philosophy, Universidad Peruana Cayetano Heredia, Av. Honorio Delgado 430, Lima, Peru; 2 Laboratory of Endocrinology and Reproduction, Universidad Peruana Cayetano Heredia, Av. Honorio Delgado 430, Lima, Peru; 3 National Meteorology and Hydrology Service (SENAMHI), Deputy Director of Evaluation of the Atmospheric Environment, Jr. Cahuide 785, Lima, Peru; 4 Atmospheric Pollution Research Group, Professional Career of Environmental Engineering, Universidad Nacional Tecnológica de Lima Sur, Sector 3 Grupo 1A 03 - Cercado (Av. Central y Av. Bolivar), Lima, Peru

**Keywords:** Air pollution, Social distancing, Particulate matter, Long-term exposure, Fatality rate

## Abstract

**Background:**

Coronavirus disease 2019 (COVID-19) originated in the People’s Republic of China in December 2019. Thereafter, a global logarithmic expansion of cases occurred. Some countries have a higher rate of infections despite the early implementation of quarantine. Air pollution might be related to high susceptibility to the virus and associated case fatality rates (deaths/cases*100). Lima, Peru, has the second highest incidence of COVID-19 in Latin America and also has one the highest levels of air pollution in the region.

**Methods:**

This study investigated the association of levels of PM_2.5_ exposure in previous years (2010–2016) in 24 districts of Lima with cases, deaths and case fatality rates for COVID-19. Multiple linear regression was used to evaluate this association controlled by age, sex, population density and number of food markets per district. The study period was from March 6 to June 12, 2020.

**Results:**

There were 128,700 cases in Lima and 2382 deaths due to COVID-19. The case fatality rate was 1.93%. Previous exposure to PM_2.5_ (2010—2016) was associated with the number of COVID-19- cases (*β* = 0.07; 95% CI: 0.034–0.107) and deaths (*β* = 0.0014; 95% CI: 0.0006–0.0.0023) but not with the case fatality rate.

**Conclusions:**

After adjusting for age, sex and number of food markets, the higher rates of COVID-19 in Metropolitan Lima are attributable to the increased PM_2.5_ exposure in the previous years, among other reasons. Reduction in air pollution from a long-term perspective and social distancing are needed to prevent the spread of virus outbreaks.

**Supplementary Information:**

The online version contains supplementary material available at 10.1186/s12889-021-11232-7.

## Background

Coronavirus disease 2019 (COVID-19), which is caused by severe acute respiratory syndrome corona virus 2 (SARS-CoV-2), was first reported in December 2019 in Wuhan in Hubei Province of the People’s Republic of China [1]. On March 11, 2020, due to the global logarithmic expansion of cases, COVID-19 was declared a pandemic by the World Health Organization (WHO) [2]. On March 6, 2020, the Peruvian president announced the first case of COVID-19 infection in Lima – an imported case of a Peruvian national who had returned from recent travel to France, Spain, and the Czech Republic [3]. On March 11, 2020, the president declared a general quarantine with social distancing interventions, including closures of all educational institutions (i.e., schools and universities) in Peru; on March 16, 2020, a national emergency was declared [3].

Three months after the declaration of the emergency and strict measures of social isolation, rates of infection were extremely higher in the metropolitan area of Lima than in the rest of the country. As of June 12, 2020, there were 128,700 (58.3%) COVID-19 cases in Lima and 92,049 (41.7%) in the rest of the country. Several reasons have been suggested to explain the high incidence of COVID-19 and the inability to reduce the scaling up of the COVID-19 outbreak.

A similar situation with a high incidence of the outbreak in one specific region that was different from that in other regions has been described in Italy, where the highest incidence of COVID-19 was observed in the northern part of the country [4, 5].

Several studies have explored the association between viral transmission and the COVID-19 mortality rate with environmental factors, including air pollution [6, 7].

One explanation of the higher rate of viral infection is that the susceptibility of the population to the virus is predetermined by exposure to air pollutants in previous years [6]. Similarly, a higher rate of infection with COVID-19 in northern Italy was apparently related to the highest levels of air pollution in that part of the country [8]. It is suspected that individuals with chronic PM_2.5_ exposure have progressive and chronic inflammation of the respiratory tract and are more prone to severe respiratory diseases after viral infections [4]. Indeed, epidemiological evidence associates chronic PM_2.5_ exposure with health problems [9–11]. Nonetheless, in a recent study, researchers did not observe an association between PM_2.5_ exposure and COVID-19 transmission during the first months of the pandemic [12].

Lima is one of the most polluted cities in Latin America [13], and it is possible that long-term exposure to air pollutants may increase infection susceptibility of individuals to different external agents, including bacteria and viruses [14–16]. In the city of Lima, PM_2.5_ concentrations vary by seasonality based on meteorological conditions. In late autumn and winter (lower temperatures), gas–particle conversion processes increase environmental PM_2.5_ concentrations [17]. Another possibility is that the virus may be adhered onto particulate matter (PM), which then acts as a vector for spread, extending the persistence of viral particles in the air and thereby favoring “indirect” transmission in addition to direct spread (individual to individual) [5].

Based on these arguments, it has been suggested that the reduction in air pollution that occurred secondary to the shutting down of national and international transportation might reduce spread of the disease [18, 19] and thus may explain the decreased incidence of COVID-19.

Experience in Peru seems to demonstrate that the reduction in air pollution was unassociated with the reduction in COVID-19 cases. The daily PM_2.5_ concentrations showed a gradual decrease from March 16 and did not exceed the environmental quality standards for air that were specified by the Peruvian Ministry of the Environment. On average, a 38% decrease in PM_2.5_ was recorded (during the first 15 days of the state of emergency) for Lima compared to its historical concentrations (2015–2019) [20]. However, in the same period, cases of COVID-19 in Lima (1338 × 100,000 inhabitants) increased much more than in the rest of the country (399 × 100,000 inhabitants), representing 58% of cases countrywide.

Unlike other governments in the world, the Government of Peru declared quarantine as soon as the first case was detected. After declaration of the emergency, the level of air pollutants was greatly reduced, though new cases of COVID-19 continued to increase.

We hypothesized that air pollution might have a chronic effect that increases the susceptibility of individuals to the virus and that individuals living in locations with high air pollution in the years preceding the pandemic are at a higher risk of infection.

To evaluate this hypothesis, this study involved analysis of data from 24 districts of Lima that were characterized by different levels of PM_2.5_ exposure in the years preceding the COVID-19 outbreak. We sought to evaluate whether the values obtained in previous years were associated with the incidence and mortality rates of COVID-19.

The primary objective was to determine whether long-term exposure to different PM_2.5_ concentrations is associated with the number of cases, deaths and case fatality rates of COVID-19. Then, we determined whether this association was modified by age, sex, and number of food markets per district. This study is different from numerous other papers that discuss the association between current pollution levels and pandemic intensity.

## Methods

This cross-sectional study assessed the association between air pollution and the high incidence of COVID-19 in Lima and was undertaken as an ecological study involving analysis of two secondary databases. We assessed data for COVID-19 cases and deaths that occurred in the Lima metropolitan area until June 12, 2020. We comparatively analyzed data on COVID-19 using the estimated daily levels of PM_2.5_ measured in the years between 2012 and 2016 [21].

Previously, Vu et al. [21] employed the total number of daily ground PM_2.5_ measurements obtained from ten monitors in the Servicio Nacional de Meteorología e Hidrología del Perú (SENAMHI) network and six Johns Hopkins University monitoring sites from March 2010 through December 2016. Data accounted for 19% of the days where any monitor recorded a measurement on a given day. Consequently, daily ambient PM_2.5_ concentrations from March 2010 to December 2016 were estimated by a random forest (RF) model developed by Vu et al. [21]. The RF model calibrated satellite aerosol optical depth (AOD), meteorological parameters from chemical transport models, and land use variables with available ground measurements from the two monitoring networks (SENAMHI and Johns Hopkins). For this study, we obtained the annual mean value of PM_2.5_ from 2012 and 2016, and then we obtained an average value of these five-year periods (Supplementary Table 1). We also included data on temperature and relative humidity.

The district was taken as the unit of analysis. In this study, 24 districts of Lima were included: Ate, Barranco, Carabayllo, Chorrillos, Comas, El Agustino, Independencia, La Molina, La Victoria, Lima, Lince, Los Olivos, Puente Piedra, Rímac, San Borja, San Isidro, San Juan de Lurigancho, San Juan de Miraflores, San Luis, San Martín de Porres, Santiago de Surco, Surquillo, Villa el Salvador, and Villa María del Triunfo. These districts included a total population of 7,029,238 inhabitants and a population density of 241,623 people per square kilometer.

PM_2.5_ data were obtained from the National Meteorology and Hydrology Service of Peru (SENAMHI), which has ten stations that record PM_2.5_ daily concentrations in Lima. Data were obtained through an agreement between SENAMHI and Universidad Peruana Cayetano Heredia as part of the Regional GEOHealth Hub centered in Peru. Details of the construction of the database were published previously [21]. Other pollutants were not included because there were insufficient district-level data for analysis.

Data on the population and surface areas of the provinces and elevation of the capitals of the provinces in Peru were obtained from the Peruvian Center for planning website (https://www.ceplan.gob.pe/informacion-sobre-zonas-y-departamentos-del-peru/).

In the 24 districts, there were 94,273 COVID-19 cases and 1987 deaths. The case fatality rate due to COVID-19 was 2.58% in Peru and 1.93% in Lima. There were 948 food markets in the 24 districts of Lima (Peru). Data from the COVID-19 database of the Open Data website of Peru (https://www.datosabiertos.gob.pe/group/datos-abiertos-de-covid-19) were used to analyze the information on COVID-19 deaths and COVID-19 cases.

### Statistical analysis

Data were managed in the MS Excel 2016 program. The STATA v14.0 statistical package (StataCorp, College Station, TX, USA) was used for analysis and ArcGIS 10.5 for map construction. First, the average amounts of PM_2.5_ were calculated for each district from 2012 to 2016 (Supplementary Table 2). Data on age at infection and at death are presented as the mean ± standard deviation, and differences between the pair of means were calculated by Student’s *t*-test. The number of COVID-19 cases and deaths by each district was evaluated overall and by sex.

We explored distinct linear models to assess the relationship of the reported cases and deaths by sex and the sex ratio at the district level in the province of Lima.

As a secondary objective, we assessed the association between the number of food markets per district and cases and deaths due to COVID-19. Data from the number of food markets in each district were obtained from a survey in 2016 (www.inei.gob.pe/media/MenuRecursivo/publicaciones_digitales/Est/Lib1447/libro.pdf).

The associations between COVID-19 cases, deaths due to COVID-19 and case fatality rates (deaths/cases of COVID-19*100) and previous PM_2.5_ exposure were evaluated by using linear regression controlled by age, sex, population density and number of food markets per district. Ambient temperature and relative humidity were also controlled in the analysis. Statistical significance was considered at *p* < 0.05.

## Results

By June 12, 2020, Peru had 220,749 COVID-19 cases and 6308 deaths. Lima had 128,700 COVID-19 cases and 2382 deaths. Among all identified COVID-19 cases and deaths, 59.1% (*N* = 130,462) and 71.1% (*N* = 4485), respectively, were men. The number of deaths in the 24 districts studied represents 94.8% of all COVID-19-related deaths in Lima.

The COVID-19 case fatality rate was 2.58% nation-wide and 1.93% for Lima. The mean age at infection was 20 years younger than the age at death due to COVID-19. The patterns in the country and Lima Metropolitan area were similar. Women became infected and died at later ages than men (Table 1).

**Table 1:** Sex-stratified differences in age at COVID-19 confirmation and at death due to COVID-19 at the national level and in the province of Lima

Statistical variables	Age among men (years)	Age among women (years)
Cases at the country level	42.93 ± 16.88 (*n* = 130,333)	43.17 ± 1 7.69* (*n* = 90,256)
Cases at the Lima level	43.01 ± 16.90 (*n* = 72,992)	43.67 ± 17.96* (*n* = 50,613)
Deaths at the country level	64.51 ± 13.85 (*n* = 4047)	66.68 ± 14.91* (*n* = 1643)
Deaths at the Lima level	64.43 ± 14.12 (*n* = 1695)	67.37 ± 14.47* (*n* = 687)

Data are the mean ± SD

*p < 0.01, with regard to values for men

**Fig. 1:**
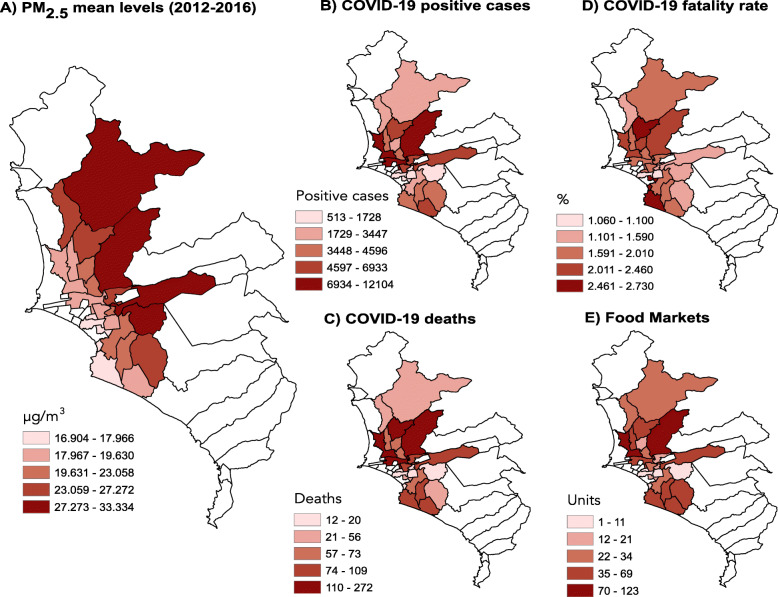
Distribution of air pollution and COVID-19 cases in Lima. **A** Particulate matter ≤2.5 μm (PM_2.5_), **B** incidence of COVID-19 cases, **C** incidence of COVID-19 deaths, **D** COVID-19 fatality rate (Deaths/Cases*100), and **E** abundance of food markets. Environmental data are expressed as μm/m^3^ and refer to the mean values for 2012–2016. The distribution data for COVID-19 were obtained from the Ministry of Health of Peru (COVID-19 data updated until June 12, 2020)

The mean concentrations of PM_2.5_ from 2012 to 2016 for the 24 districts evaluated in this study are shown in Fig. 1A, whereby the lighter red zone refers to lower levels of PM and the darkest tone to the highest level.

The incidence of COVID-19 cases and deaths are shown in Fig. 1B and C, respectively. The highest incidence of cases and deaths occurred in districts located to the north of the city. The COVID-19 case fatality rates (Deaths/Cases*100) in Lima are shown in Fig. 1D, and the abundance of food markets per district is illustrated in Fig. 1E. Moreover, the largest numbers of markets were found in districts with the highest COVID-19 incidence of cases and deaths (northern part of the city).

**Fig. 2:**
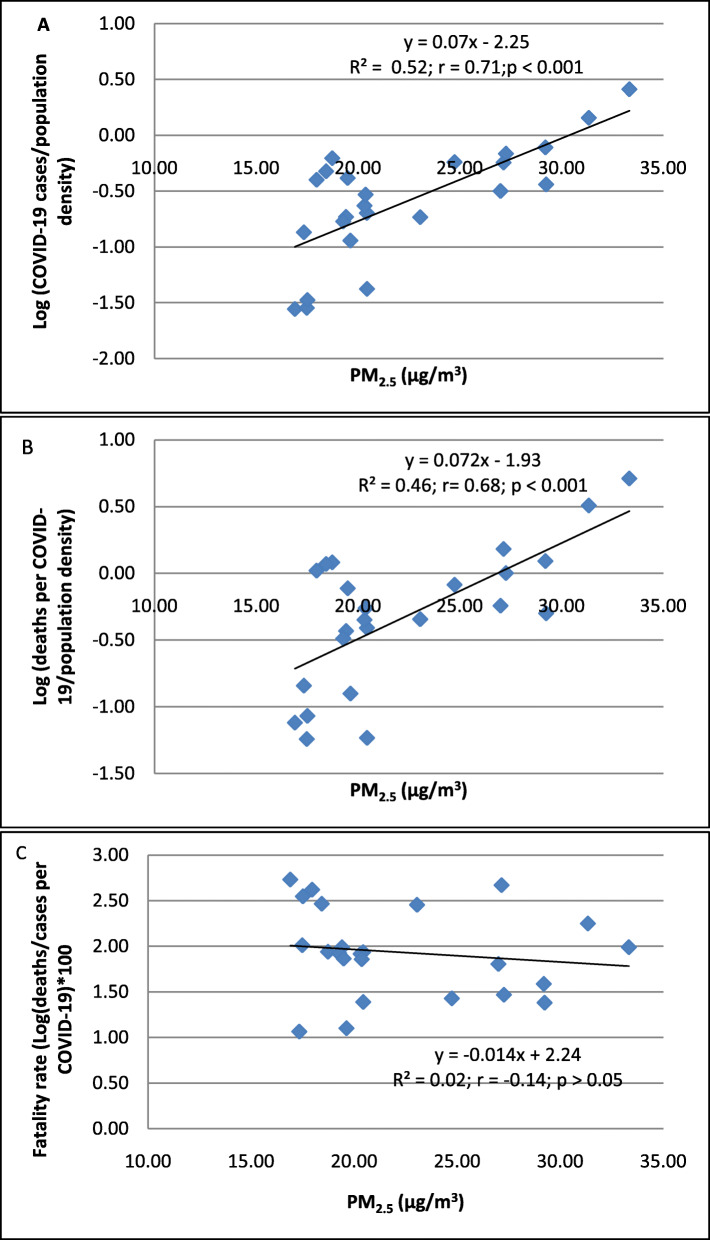
The association between PM_2.5_ and Log (cases of COVID-19/population density) (**A**), Log (deaths per COVID-19/population density) (**B**), and fatality rates *100 (**C**) in 24 districts of Metropolitan Lima. The population of the district was ascertained from the values reported in the census of 2017. This number was not corrected based on the estimation of growth

Higher PM_2.5_ levels were associated with a higher incidence of COVID-19 cases and deaths in 24 districts of Metropolitan Lima (Fig. 2A and B). However, the case fatality rate did not increase with increasing levels of PM_2.5_ (Fig. 2C).

**Fig. 3:**
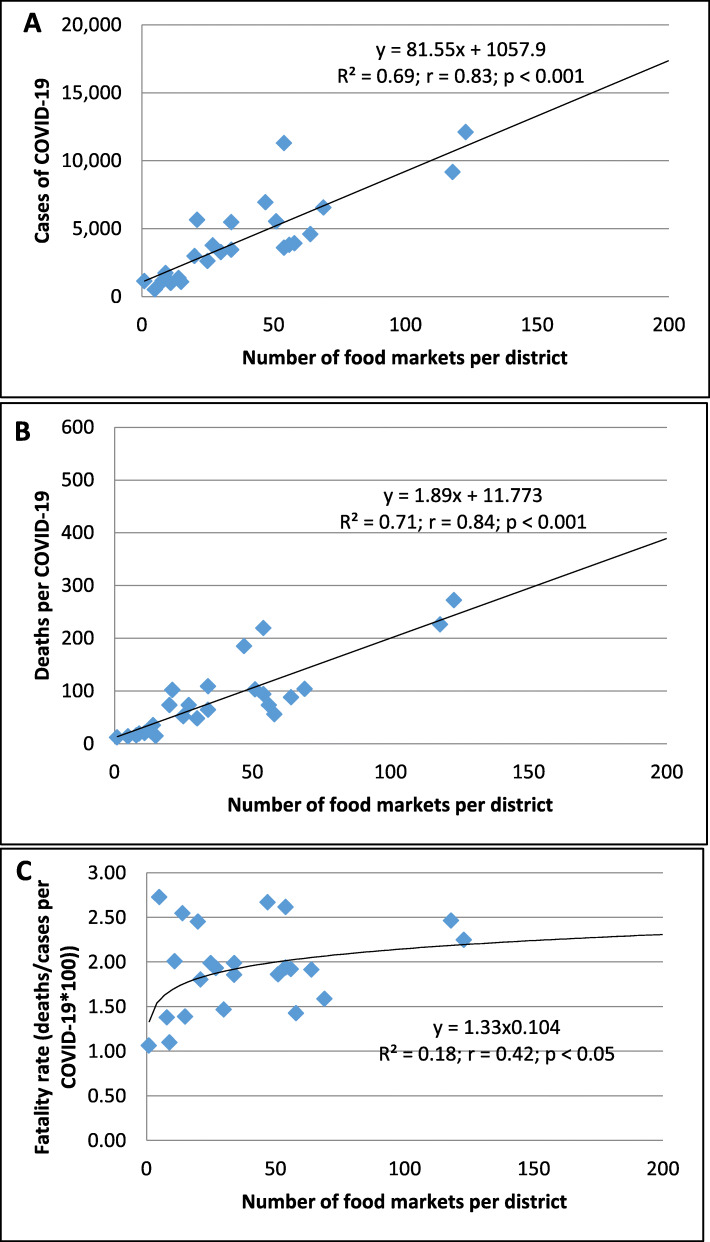
The association between the number of food markets per district and cases of COVID-19 (**A**), deaths per COVID-19 (**B**), and fatality rates *100 (**C**) in 24 districts of Metropolitan Lima

Similarly, a higher number of food markets was linearly associated with a higher number of COVID-19 cases and mortality (*p* < 0.01, Fig. 3A and *p* < 0.01, Fig. 3B, respectively).

The association between the number of food markets and COVID-19 cases and mortality persisted when cases (*R*^*2*^ = 0.25; *r* = 0.49; *p* < 0.01) and deaths (*R*^*2*^ = 0.33; *r* = 0.58; *p* < 0.01) were adjusted by population density. Furthermore, the case fatality rate was associated with an increased number of food markets (*p* < 0.05; Fig. 3C).

However, the number of food markets per district did not correlate with PM_2.5_ (μg/m^3^) (Y = 0.033x + 21.05; *R*^*2*^ = 0.04; *r* = 0.21; *p* > 0.05). This suggests that PM_2.5_ and the number of food markets were independently associated with the spread of COVID-19. Moreover, a higher number of food markets was associated with an increased case-fatality rate, whereas a higher PM_2.5_ level did not increase the fatality rate (data not shown).

In general, the higher the number of food markets per district was, the higher was the incidence of COVID-19/population density (y = 0.03 × ^0.63^; *r* = 0.58; *p* < 0.01). Additionally, the higher the number of food markets per district was, the higher was the COVID-19 mortality/population density*100 (Y = 0.04 × ^0.73^; *r* = 0.66; *p* < 0.001; data not shown).

**Fig. 4:**
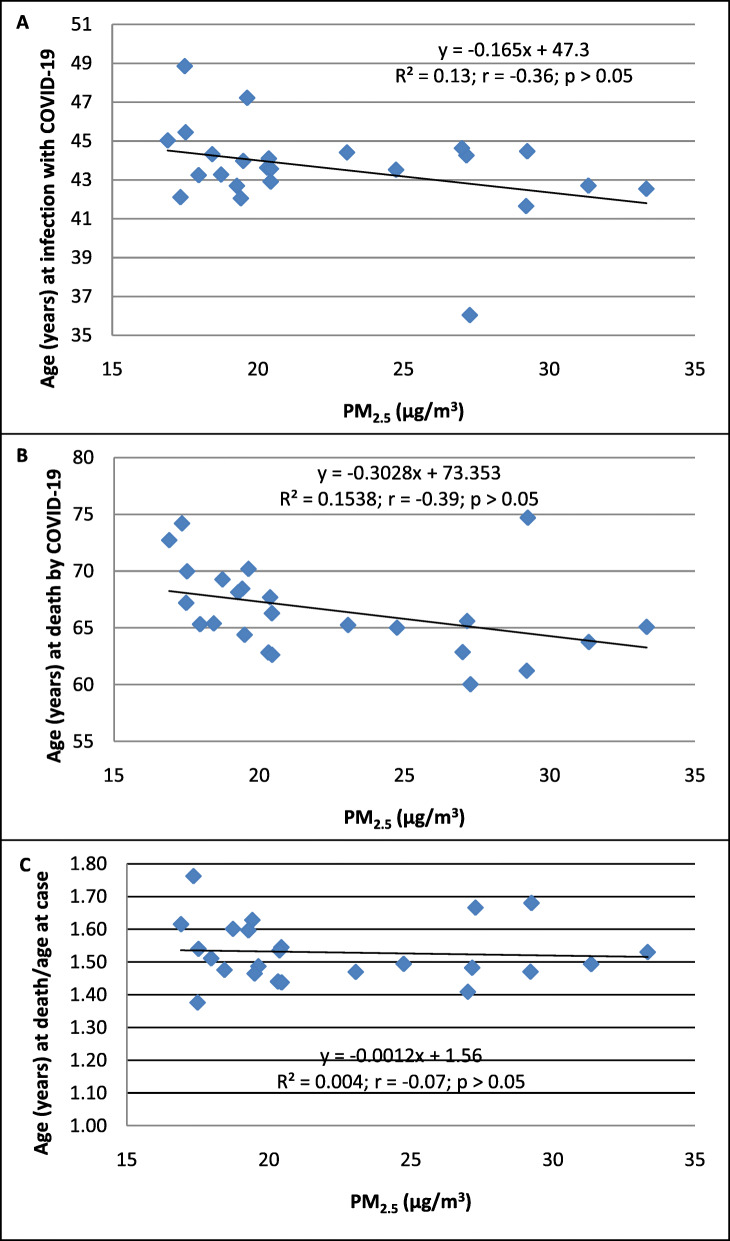
The association between PM_2.5_ (μg/m^3^) and age (years). **A** Age at COVID-19 diagnosis, **B** age at death due to COVID-19, and **C** age at death/age at confirmation of COVID-19 in 24 districts of Metropolitan Lima. The population of the district was ascertained from the values reported in the census of 2017. This number was not corrected based on the estimation of growth

Increasing PM_2.5_ levels were not associated with specific age at infection, death, or age at death/age at infection in COVID-19 (*p* > 0.05; Fig. 4A, B, and C).

GLM analysis showed that increasing levels of PM_2.5_ (μg/m^3^) in the previous years (2012–2016) were associated with a higher incidence of COVID-19 in the 24 districts of Lima included in the study. This was also observed after adjusting for the sex ratio, age at onset, and number of food markets per district (Table 2). Table 3 shows that PM_2.5_ was associated with death/population density after controlling for age, sex and number of food markets. In contrast, after controlling for different variables, the case fatality rate (deaths/cases*100) was not associated with increasing levels of PM_2.5_ (Table 4). Additionally, environmental temperature and relative humidity were not associated with cases of COVID-19/density population or with death due to COVID-19 (Supplementary Table 3), whereas temperature was inversely associated with CFR *100 (Supplementary Table 3).

**Table 2:** Association between COVID-19 case/population density and previous PM_2.5_ concentrations in 24 districts of Lima

COVID-19 case/population density	Crude coefficient	95%CI		Adjusted coefficient	95%CI	
PM_2.5_	0.083**	0.050	0.115	0.070**	0.034	0.107
Sex ratio	−3.133*	−5.329	−0.937	−2.157	−5.127	0.812
Age	−0.080	−0.181	0.021	0.047	−0.064	0.158
Food markets	1.269	−0.259	2.796	0.242	−0.973	1.457

The model was adjusted for PM_2.5_ level, sex ratio (female cases: male cases), and food markets (number of food markets per district and age [years] at the COVID-19 diagnosis). Data for the PM_2.5_ level (μg/m^3^) correspond to the average data per district obtained daily from 2012 to 2016

*p < 0.05; **p < 0.01

**Table 3:** Association between COVID-19 death/population density and previous PM_2.5_ concentrations in 24 districts of Lima

COVID-19 deaths/population density	Crude coefficient	95%CI		Adjusted coefficient	95%CI	
PM_2.5_	0.0016**	0.0008	0.0023	0.0014*	0.0006	0.0023
Sex ratio	0.019	−0.005	0.044	0.009	−0.010	0.029
Age	−0.001	−0.002	0.000	0.000	−0.001	0.002
Food markets	0.029	−0.002	0.060	0.024	−0.007	0.055

The model was adjusted for PM_2.5_ level, sex ratio (female deaths: male deaths), and food markets (number of food markets per district and age [years] at the moments of death by COVID-19). Data for the PM_2.5_ level (μg/m^3^) correspond to the average data per district obtained daily from 2012 to 2016

*p < 0.05; **p < 0.01

**Table 4:** Association between the COVID-19 case fatality rate and previous PM_2.5_ concentrations in 24 districts of Lima

COVID-19 fatality rate	Crude coefficient	95%CI		Adjusted coefficient	95%CI	
PM_2.5_	− 0.014	− 0.056	0.029	0.01	−0.047	0.068
Sex ratio	−0.303	−2.569	1.963	−1.956	−5.207	1.293
Age	0.043	−0.049	0.135	0.035	−0.039	0.11
Food markets	1.082	−0.257	2.422	0.0054	−0.001	0.012
Population density	0.00001	−6.21e-^06^	.000035	0.00003	−2.60e-^06^	0.00006

The model was adjusted for PM_2.5_ level, sex ratio (female cases: male cases), age [years], and food markets (number of food markets per district and population density at the COVID-19 diagnosis). Data for the PM_2.5_ level (μg/m^3^) correspond to the average data per district obtained daily from 2012 to 2016

*p* > 0.05

## Discussion

This study showed that exposure to high levels of PM_2.5_ in the years preceding the COVID-19 pandemic is associated with a higher incidence of COVID-19 infection and mortality due but not the case fatality rate (Deaths/cases*100) in Metropolitan Lima, a city that is considered one of the most polluted in Latin America [13]. The findings suggest that the current incidence of COVID-19 is associated with chronic exposure to air pollution. Furthermore, the association was maintained after controlling for population density, age, sex and number of food markets.

This is an important finding that explains why cases of COVID-19 increased in Lima despite Peru having one of the earliest COVID-19 lockdowns in Latin America, applying a quarantine, with many activities closed (schools, universities, churches, ban of public events), soon after the first case of COVID-19 was detected.

In Peru, some specific factors might contribute to the spread of COVID-19 during quarantine. These include the easy availability of food markets, banks, and public transport. In Peru, food markets remained open during the quarantine period to ensure food availability for the population. We believe most of the contagions at this time were due to the congregation of people in these food markets. According to the findings of this study, there were higher numbers of cases and deaths in districts where there were more markets. This may explain the high spread of COVID-19 in Lima, in a situation in which individuals are susceptible to the virus by previous exposure to air pollutants.

A previous systematic analysis showed that the incidence of and risk of morbidity and mortality from COVID-19 increase with chronic and acute exposure to air pollution, particularly to PM (PM_2.5_ and PM_10_) and nitrogen dioxide [8, 22–24].

Values of PM_2.5_ ranged from 14.50 to 41.69 μg/m^3^ for all districts, which were all higher than the annual mean value declared by the WHO (10 μg/m^3^). Thus, as it was previously shown that PM_2.5_ is associated with an increasing risk for respiratory infectious diseases [16], the present situation with the spread of COVID-19 demonstrates an increasing trend. Although we assessed the association with long-term PM_2.5_ exposure, the impact of short-term exposure needs to be addressed in further research.

Analysis of the distribution of COVID-19 cases worldwide demonstrates remarkable asymmetry with respect to countries/regions [25, 26]. This is exactly the pattern that we observed in Metropolitan Lima. For the 24 districts assessed, those with higher PM_2.5_ concentrations during 2012–2016 showed a higher COVID-19 incidence than did those with lower concentrations of pollutants (Supplementary Table 2). Nationwide, 220,749 cases and 6308 deaths due to COVID-19 have been reported since the first case. Lima has 58.3% of the national cases and 38% of deaths, though Lima represents only 28% of the total Peruvian population.

Human pathogenic coronaviruses, which include SARS-CoV-2, which is responsible for COVID-19, bind to target cells through the angiotensin-converting enzyme 2 (ACE2) receptor, which is expressed by epithelial cells of the lung, intestine, kidney, and blood vessels [27]. It is possible that PM_2.5_ induces elevated ACE2 activity.

Additionally, active cigarette smoking upregulates ACE-2 expression in the lower airways, which may partially explain the increased risk of severe COVID-19 in these populations [28]. If air pollutants act similarly to the agents released during smoking, it is probable that individuals with chronic exposure to these compounds would have low ACE-2 activity or greater susceptibility to the infection [29].

This study in Metropolitan Lima that used PM_2.5_ data from 2012 and 2016 for 24 districts showed that the case fatality rate did not increase with increasing values of PM_2.5_. This is an interesting finding because crude analysis of the data showed a higher rate of death when PM_2.5_ concentrations are higher. However, when the data were calculated as the number of COVID-19 deaths/number of cases (case-fatality rate), there was no observable association with PM_2.5_. Regardless, the results suggest that PM_2.5_ does not affect the case fatality rate. This agrees with data observed in New York after short-term exposure to air pollutants [6].

The national COVID-19 case fatality rate was 2.58%, and the rate was 1.93% for Lima. The higher rate in the rest of Peru with respect to Lima may reflect deficiencies in the healthcare system in the different provinces. In addition, the COVID-19 case fatality rate was higher in men and increased with age, thus confirming previous results [30].

On average, the age at infection was approximately 42 years in Peru, and it is possible that the age at infection will decrease after the end of quarantine. This is because more young people will be exposed.

At national and province levels (Lima), age at infection was 20 years younger than age at death due to COVID-19. This higher mortality risk for older people has already been reported [31, 32]. In our study, the association between older age and COVID-19 mortality risk was unaffected by increasing levels of PM_2.5_, suggesting that the factors that explain the higher mortality risk with age are independent of PM_2.5_ exposure. In fact, PM_2.5_ did not modify the COVID-19 case fatality rate in Lima.

Lima has an average elderly population of 13% and under 19 years of age population of 22%. Districts of the suburbs (Ate, Puente Piedra, Carabayllo, VES and VMT) have the lowest elderly population and therefore more young people. The largest population (19–64 years) is similar in all districts, and those who move around the city for work, shopping, entertainment are the most exposed to infection [33].

There is evidence of the role of PM pollutants in COVID-19 transmission. PM_2.5_ and other small PM can act as disease vectors and facilitate airborne transmission of viable virus particles, which have been implicated in the spread of measles and SARS [34].

Social distancing is an important preventable measure to decrease the spread of COVID-19. This was demonstrated in 28 European countries, where the most likely point of change during the COVID-19 epidemic showed a dose-response association of the observed flattening of the epidemic curve with an increasing social distancing index (SDI). Countries in the highest SDI quartile achieved a statistically significant decline in the incidence and prevalence of the disease [34].

According to a recent report, social distancing measures that were adopted by the population in Brazil appeared to be effective, particularly when implemented in conjunction with the isolation of cases and quarantine of contacts [35].

In numerical simulations, three scenarios were compared in a city within Brazil: first was the vertical distancing policy, where only older people were distanced; the second involved the horizontal distancing policy where all age groups adhered to social distancing; and the third involved a control scenario in which no intervention was undertaken to distance people. Horizontal distancing, if applied with the same intensity in all age groups, significantly reduced the total number of infected people by “flattening the disease growth curve”; conversely, vertical distancing or no distancing did not have this effect [36].

Doubtless, social distancing measures appear to be the most effective intervention to slow the disease spread of COVID-19. Although studies unanimously confirm the mitigating effect of social distancing on disease spread, the reported effectiveness varies from 10% to a more than 90% reduction in the number of infections [34]. Changes in mobility in public places, such as retail and recreation centers (e.g., restaurants, cafes, theatres), grocery stores and pharmacies, transit hubs (e.g., airports, bus stations, subways), and parks are the most important determinants of the disease transmission rate [37].

It is well known that the highest risk of COVID-19 occurs prior to symptom onset. A recent paper provides evidence of the effectiveness of mask use, disinfection, and social distancing in the prevention of COVID-19 [38].

In this study, the number of markets was unrelated to the PM_2.5_ concentration, which suggests that both factors are independently associated with the spread of COVID-19. Moreover, after controlling for different variables, including the number of food markets, PM_2.5_ remained associated with the number of cases of COVID-19.

From a long-term perspective, a reduction in air contamination should be considered a part of the integrated approach for sustainable development, human health protection, and reducing the *spread* of a disease during *an outbreak*, *epidemic*, or *pandemic*. Nevertheless, although reducing air pollution is important to reduce morbidity and mortality due to different diseases, the findings of this study also suggest the importance of social isolation to reduce the incidence of COVID-19 [39]. The magnitude of contagion in food markets is an example that policies aimed at reducing crowding should be important for preventing the spread of COVID-19.

We did not find an association between environmental temperature and COVID-19 cases or deaths. However, CFR was inversely related to increased temperature. Other authors have also observed that temperature may not be a determinant inducing COVID-19 spread [40, 41]. Another study indicates that lower temperature may increase COVID-19 transmission, but there is no evidence that temperature affects the case fatality rate [42]. Humidity has been negatively associated with COVID-19-related death [43]. Our study, however, showed no association between humidity and COVID-1 death. Further studies are required to clarify different results between countries.

The limitations of this study are a lack of data for the number of people attending food markets during the quarantine period. We were also unable to obtain data for people visiting banks and using public transportation within the same period. Data regarding for social distancing and the use of masks are also lacking.

The study makes a significant contribution to the literature because the findings indicate that past exposure to higher PM_2.5_ levels is associated with a higher incidence of COVID-19 disease and mortality. However, the case fatality rate did not increase with increases in PM_2.5_ levels. This study is different from other papers that discuss association between current pollution levels and pandemic intensity.

The findings of this study are generalizable to regions with similar population densities and PM_2.5_ levels in the setting of respiratory epidemics or future pandemics. The findings will be a support tool for decision-making regarding the country’s health policy, as a study in which PM_2.5_ is associated based on district, age, sex, and food markets with the number of COVID-19 cases and mortality will allow us to rethink the measures used by the Peruvian government and other countries characterized by high air pollution.

In conclusion, the present study demonstrates that higher rates of spread of COVID-19 in Metropolitan Lima (Peru) are associated with previous long-term PM_2.5_ exposure. Men and older people were at higher risk of death due to COVID-19. Reduction in air pollution from a long-term perspective and social distancing are needed to prevent the spread of virus outbreaks. These results should be considered by officers of the government to be applied in health policies aimed at preventing or reducing epidemic viral spread. The strategies taken to confront the pandemic should also consider previous environmental indicators to intensify efforts in areas with higher air pollution.

## Supplementary Information

**Additional file 1.**

## Data Availability

The datasets generated and/or analyzed during the current study are available in the following repositories: **• Data of food markets:** INEI repository:www.inei.gob.pe/media/MenuRecursivo/publicaciones_digitales/Est/Lib1447/libro.pdf **• Data related to COVID-19: MINSA repository:**https://www.datosabiertos.gob.pe/dataset/casos-positivos-por-covid-19-ministerio-de-salud-minsa https://www.datosabiertos.gob.pe/dataset/fallecidos-por-covid-19-ministerio-de-salud-minsa **• Environmental data:** Data were obtained as a part of an agreement between SENAMHI and Universidad Peruana Cayetano Heredia as part of the Regional GEOHealth Hub centered in Peru. The datasets of predicted PM_2.5_ concentrations at 1-km^2^ spatial resolution in Lima-Peru from 2010 to 2016 used during the study are available from the corresponding author upon reasonable request.

## Abbreviations

ACE2: Angiotensin-converting enzyme 2
CI: Confidence interval
COVID-19: Coronavirus disease 2019
°C: Centigrade degree
GEOHealth: Global Environmental and Occupational Health
GLM: Generalized linear model
Log: Logarithm with base 10
P: Probability
PM_2.5_: Fine particulate matter
PM_10_: Solid or liquid particles with diameter varying between 2.5 and 10 μm
R^2^: Coefficient of determination
r: Coefficient of Pearson
SARS: Severe acute respiratory syndrome
SARS-CoV-2: Severe acute respiratory syndrome coronavirus 2
SD: Standard deviation
SDI: Social distancing index
SENAMHI: National Service of Meteorology and Hydrology of Peru
SIDISI: Decentralized Information System and Monitoring of Research
WHO: World Health Organization
