# Supplementary material for: Association between air pollution in Lima and the high incidence of COVID-19: findings from a post hoc analysis
Source: BMC Public Health. 2021 Jun 16;21:1161. doi: 10.1186/s12889-021-11232-7 (PMC8208068; doi:10.1186/s12889-021-11232-7)
Supplement: Supplementary file 1 — Additional file 1. [file 12889_2021_11232_MOESM1_ESM.docx]

**Supplementary Table 1.** Annual PM_2.5_ estimated (*µg/m3*) average by district.

| Districts | 2012 | 2013 | 2014 | 2015 | 2016 | Total average |
| --- | --- | --- | --- | --- | --- | --- |
| Ate | 27.99 | 28.88 | 28.36 | 28.65 | 32.16 | 29.208 |
| Barranco | 17.36 | 17.16 | 17.1 | 16.18 | 16.72 | 16.904 |
| Carabayllo | 33.24 | 33.37 | 33.08 | 31.06 | 35.92 | 33.334 |
| Chorrillos | 18.23 | 17.88 | 17.94 | 17.92 | 17.86 | 17.966 |
| Comas | 26.65 | 27.25 | 26.54 | 26.4 | 28.94 | 27.156 |
| El Agustino | 26.24 | 26.96 | 26.11 | 26 | 29.7 | 27.002 |
| Independencia | 22.42 | 23.03 | 22.88 | 22.62 | 24.34 | 23.058 |
| La Molina | 28.42 | 28.88 | 28.44 | 28.59 | 31.89 | 29.244 |
| La Victoria | 18.91 | 19.1 | 19.48 | 19.29 | 20.29 | 19.414 |
| Lima | 18.5 | 18.55 | 18.73 | 18.62 | 19.28 | 18.736 |
| Lince | 17.73 | 17.72 | 17.61 | 17.19 | 17.19 | 17.488 |
| Los Olivos | 19.01 | 19.02 | 19.27 | 19.19 | 19.9 | 19.278 |
| Puente Piedra | 26.84 | 27.33 | 26.69 | 26.49 | 29.01 | 27.272 |
| Rimac | 19.64 | 19.86 | 20.47 | 20.54 | 21.68 | 20.438 |
| San Borja | 19.08 | 19.16 | 19.78 | 19.5 | 20.63 | 19.63 |
| San Isidro | 17.54 | 17.46 | 17.47 | 17.11 | 17.13 | 17.342 |
| SJL | 30.45 | 31.85 | 30.27 | 30.06 | 34.09 | 31.344 |
| SJM | 19.99 | 19.9 | 20.24 | 20.37 | 21.1 | 20.32 |
| San Luis | 19.65 | 19.98 | 20.39 | 20.41 | 21.8 | 20.44 |
| SMP | 18.34 | 18.44 | 18.42 | 18.26 | 18.71 | 18.43 |
| Surco | 20.05 | 20.08 | 20.36 | 20.25 | 21.13 | 20.37 |
| Surquillo | 17.68 | 17.63 | 17.74 | 17.29 | 17.25 | 17.51 |
| VES | 19.37 | 19.31 | 19.32 | 19.68 | 19.8 | 19.49 |
| VMT | 24.35 | 24.35 | 24.51 | 24.33 | 26.16 | 24.74 |
| Annual average | **21.99** | **22.21** | **22.13** | **21.92** | **23.45** | **22.34** |

SJL: San Juan de Lurigancho; SJM: San Juan de Miraflores; SMP: San Martin de Porres; VES: Villa El Salvador; VMT: Villa Maria del Triunfo.

**Supplementary Table 2** Numbers of confirmed cases and deaths due to COVID-19, the COVID-19 fatality rate, and mean values of the PM_2.5_ concentration in the 2012-2016 period per district in Lima

| Districts | COVID-19 cases | COVID-19 deaths | Fatality rate*100 | PM_2.5_  (µg/m^3^) | Population density (people/square meters) | Food markets |
| --- | --- | --- | --- | --- | --- | --- |
| Ate | 6,556 | 104 | 1.58 | 29.208 | 8433 | 69 |
| Comas | 6,933 | 185 | 2.67 | 27.156 | 11021 | 47 |
| San Juan de Lurigancho | 12,104 | 272 | 2.25 | 31.344 | 8674 | 123 |
| El Agustino | 5,654 | 102 | 1.80 | 27.002 | 15574 | 21 |
| Puente Piedra | 3,274 | 48 | 1.46 | 27.272 | 5241 | 30 |
| Barranco | 513 | 14 | 2.72 | 16.904 | 18421 | 5 |
| San Juan de Miraflores | 4,596 | 88 | 1.91 | 20.32 | 17342 | 64 |
| Villa el Salvador | 5,528 | 103 | 1.86 | 19.496 | 13594 | 51 |
| Carabayllo | 2,615 | 52 | 1.99 | 33.334 | 1015 | 25 |
| Chorrillos | 3,590 | 94 | 2.62 | 17.966 | 8992 | 54 |
| Villa Maria del Triunfo | 3,925 | 56 | 1.43 | 24.742 | 6828 | 58 |
| La Victoria | 5,482 | 109 | 1.99 | 19.414 | 29482 | 34 |
| Lima | 11,296 | 219 | 1.94 | 18.736 | 18168 | 54 |
| Lince | 995 | 20 | 2.01 | 17.488 | 34931 | 11 |
| San Martin de Porres | 9,169 | 226 | 2.46 | 18.434 | 19261 | 118 |
| Rimac | 3,775 | 73 | 1.93 | 20.438 | 18724 | 27 |
| San Isidro | 1,129 | 12 | 1.06 | 17.342 | 8339 | 1 |
| Surco | 3,447 | 64 | 1.86 | 20.374 | 11698 | 34 |
| Los Olivos | 3,800 | 73 | 1.92 | 19.278 | 22486 | 56 |
| San Luis | 1,081 | 15 | 1.39 | 20.446 | 25672 | 15 |
| Surquillo | 1,375 | 35 | 2.54 | 17.518 | 41010 | 14 |
| La Molina | 1,160 | 16 | 1.37 | 29.244 | 3197 | 8 |
| San Borja | 1,728 | 19 | 1.09 | 19.63 | 15146 | 9 |
| Independencia | 2,976 | 73 | 2.45 | 23.058 | 16129 | 20 |
| **Total** | **102,701** | **2,072** | **1.93** | **22.34** | **384,233** | **948** |

The total represents the sum of COVID-19 cases and deaths in all 24 districts. The mean values of the data from the 24 districts are shown for the fatality rate, levels of PM_2.5_, temperature, and humidity.

**Supplementary Table 3**. Environmental variables related to COVID-19 case/population density, COVID-19 death/population density and case fatality rate (CFR) due to COVID-19

|  | **Cases/density**  **β± Std. Err.** | **P** | **Deaths/density**  **β± Std. Err.** | **P** | **CFR*100**  **β± Std. Err.** | **P** |
| --- | --- | --- | --- | --- | --- | --- |
| **PM2.5 (µg /m^3^)** | 0.10± 0.02 | 0.000 | 0.21± 0.04 | 0.000 | 0.03±.020 | 0.161 |
| **Temperature (°C)** | -0.12 ± 0.08 | 0.124 | -0.31±0.16 | 0.074 | -0.29 ± 0.08 | 0.002 |
| **Relative humidity (%)** | 0.12 ± 0.09 | 0.201 | 0.31 ±0.19 | 0.117 | 0.16 ± 0.10 | 0.111 |
| **Constant** | -7.71±6.95 | 0.280 | -19.40± 14.55 | 0.197 | -3.90 ± 7.50 | 0.609 |
| **R^2^** | 0.64 | 0.0001 | 0.63 | 0.0002 | 0.45 | 0.0064 |

Data correspond to three multivariate analyses. Data are the coefficient of regression (β) ± standard error. P = probability. R2 = Coefficient of determination. CFR= Case fatality rate *100
